# Association between the ownership of home-based records and continuous, quality maternal and child health service utilisation: a multi-country analysis of Demographic Health Surveys from 18 low- and middle-income countries

**DOI:** 10.7189/jogh.16.04052

**Published:** 2026-02-13

**Authors:** Akiko Saito, Masahide Kondo

**Affiliations:** 1 Faculty of Health Science Technology, Bunkyo Gakuin University, Tokyo, Japan; 2 Institute of Medicine, University of Tsukuba, Ibaraki, Japan

## Abstract

**Background:**

The continuity and quality of maternal and child health (MCH) services represent significant challenges in low- and middle-income countries (LMICs). Home-based records (HBRs), including the integrated Maternal and Child Health Handbook (MCHHB), may support consistent service usage and improved care quality. We aimed to evaluate the association between HBRs and continued quality MCH care, identifying differences in associations between MCHHBs and the continuum of care and those between other HBR types and the continuum of care.

**Methods:**

We conducted a pooled analysis of Demographic and Health Surveys (DHS) from 18 LMICs, including women and their youngest eligible children for the health card module. We defined the continuum of quality MCH care using receipt of quality antenatal care (ANC), skilled birth attendance, and quality postnatal care (qPNC), with ANC and PNC measured as composite indicators. We based the HBR ownership on the DHS variable ‘Has health card’. We used multivariable logistic regressions to examine associations between HBR ownership, HBR type, and the continuum of care, adjusting for residence, maternal education, and wealth.

**Results:**

The final analysis included 89 902 samples. Ownership of HBR was associated with significantly higher odds of completing the continuum of quality MCH care compared with not owning HBR. However, no significant differences were observed between owners of MCHHB and those of other HBR types. Based on analysing the subgroups of MCH service components, the MCHHB may facilitate the provision of ANC services, such as urine and blood tests, more effectively than other HBR types.

**Conclusions:**

Owning HBR was positively associated with greater use of quality MCH services. However, no significant differences were observed for MCHHBs, despite MCHHB ownership being significantly associated with improved uptakes of urine and blood tests. Further research is needed to explore the influence of actual HBR use, provider-related factors, and variations in HBR content and type.

The continuum of maternal and child health (MCH) care is essential for the survival, health, and development of mothers and children, especially in low- and middle-income countries (LMICs), which face inadequate coverage of essential MCH services despite global efforts [1]. In 2022, the proportion of women receiving at least four antenatal care (ANC) visits with any provider was 55% in Eastern and Southern Africa and South Asia, highlighting the need to improve service utilisation [2,3].

However, the quality of care delivered during each contact is as critical as the number of visits [4,5]. Dandona and colleagues highlighted a notable discrepancy between the reported coverage of ANC and the quality-adjusted ANC visits that provide essential components [6]. Many women do not receive the recommended components of ANC, including routine assessments, interventions, and counselling [7–10]. Therefore, improving maternal and neonatal outcomes requires a high-quality continuum of care and consistent delivery of appropriate interventions across the antenatal, intrapartum, and postnatal periods.

One strategy to strengthen the continuity and quality of MCH care is the use of home-based records (HBRs) [11,12]. Introduced in the mid-1800s as a vaccination card [13], HBRs have evolved into comprehensive tools. The Maternal and Child Health Handbook (MCHHB) is an integrated HBR covering the continuum of care from pregnancy to a child’s fifth year. The MCHHB documents health service use and outcomes, provides educational information for families, and facilitates provider-client communication. It supports maternal education through provider-led counselling and self-directed learning on safe pregnancy, delivery, newborn care, and child development.

To date, over 50 countries and regions, primarily LMICs, have adopted the MCHHB [14], aligning with global health priorities such as the Sustainable Development Goals and World Health Organization (WHO) recommendations [14,15]. The integrated MCHHB was introduced in LMICs in the early 2000s through pilot programmes in Indonesia and Palestine, followed by gradual scale-up in Asia, Africa, and the Pacific during the 2010s. However, empirical evidence regarding the association between HBR ownership and the continuum of quality MCH care remains limited, and few studies have compared the effects of different types of HBRs [16]. Kitabayashi and colleagues reported that MCHHB users in Palestine had significantly higher odds of receiving quality ANC (qANC); however, their study was limited to ANC and a single-country setting [17]. Another study in rural Indonesia found that the MCHHB had positive effects on service uptake and home care practices (*e.g.* tetanus vaccination, four or more ANC visits, skilled birth attendance (SBA), Vitamin A supplementation, and exclusive breastfeeding), but did not assess the ANC or PNC quality components [18]. Therefore, we aim to examine the association between HBR ownership and the completion of a continuum of quality MCH care and compare outcomes between users of the MCHHB and of other HBR types, using pooled data from multiple LMICs collected in Demographic and Health Surveys (DHS) conducted from 2015 onward, when standardised quality-of-care indicators became available.

## METHODS

### Study design and data source

We employed a cross-sectional design using pooled data from the most DHS conducted in 18 countries: Burkina Faso (2021), Côte d'Ivoire (2021), Kenya (2022), Senegal (2019), Indonesia (2017), Timor-Leste (2015), Ethiopia (2019), Gambia (2019–2020), Guinea (2018), Liberia (2019–2020), Mali (2018), Mauritania (2019–2021), Maldives (2016–2017), Nigeria (2018), Pakistan (2017–2018), Tanzania (2022), South Africa (2016), and Zimbabwe (2015). We included all data from 2015 onward (Phase 7 and later), when the core questionnaires incorporated more standardised and comparable quality-of-care indicators for MCH across countries, and when standardised data sets containing MCH data were available [19,20].

The DHS is a nationally representative household survey collecting standardised data on MCH, including service coverage, utilisation, and care content. To examine the relationship between HBR ownership and the continuum of quality MCH care, we included countries regardless of whether they introduced the integrated MCHHB.

The DHS surveys follow a standardised sampling, questionnaire design, data collection, and coding methodology, enabling valid cross-country analyses. To ensure national representativeness, DHS employs stratified two-stage sampling: selecting enumeration areas based on each country’s sampling frame and then drawing a sample of households from each [21].

For this analysis, we used the Individual Recode file, which contains data collected from women aged 15–49 years.

### Study population

Women and their youngest children (aged 0–35 months) who were eligible for the DHS ‘Has health card’ question were included, yielding 89 902 observations across countries: Burkina Faso (n/N = 1995/5637), Côte d'Ivoire (n/N = 2608/7630), Ethiopia (n/N = 9684/30 465), Gambia (n/N = 215/628), Guinea (n/N = 1585/4278), Indonesia (n/N = 14 444/5960), Kenya (n/N = 4257/14 752), Liberia (n/N = 437/1296), Mali (n/N = 2504/5075), Mauritania (n/N = 433/1296), Maldives (n/N = 22/114), Nigeria (n/N = 19 975/50 768), Pakistan (n/N = 18 538/47 186), Senegal (n/N = 1550/4275), Timor-Leste (n/N = 89/306), Tanzania (n/N = 6332/16 831), South Africa (n/N = 3870/17 272), and Zimbabwe (n/N = 1363/3992). Finally, we excluded 20 samples with missing essential MCH service data and included 36 samples with missing age data.

### Variables

#### Dependent variables

The main outcome variable was the completion of the continuum of quality MCH maternal care, defined as combined receipt of qANC, SBA, and quality postnatal care (qPNC).

We defined qANC as receiving a blood pressure measurement, taking a urine sample, taking a blood sample, and receiving or purchasing iron tablets/syrup during pregnancy. Additionally, SBA was defined as delivery assisted by an obstetrician, doctor, general practitioner, midwife, nurse, certified birth attendant, or health officer. Finally, qPNC was defined as receiving umbilical cord examination, newborn temperature measurement, counselling on newborn danger signs, and counselling on and observation of breastfeeding within the first two days after delivery.

In addition, the analysis included individual outcomes for the number of ANC visits, qANC, SBA, and qPNC.

#### Independent variables

We used the DHS variable ‘Has health card’ as a proxy for HBR ownership. In countries where the MCHHB has been introduced [22], this card referred to the MCHHB. In other settings, it represented alternative HBR types (**Table 1**).

**Table 1:** Variable description

	Description
**Quality ANC**	Coded as 1 if all five qANC components were received; otherwise coded as 0
**SBA**	Coded as 1 if attended by any skilled health personnel (*e.g.* doctor, nurse, midwife); otherwise coded as 0
**Quality PNC**	Coded as 1 if all five qPNC components were received within two days of delivery; otherwise coded as 0
**Completion of continuum**	Defined as having qANC = 1 + SBA = 1 + qPNC = 1
**Owns MCHHB/HBR**	MCHHB/HBR present: 1 = card seen; MCHHB/HBR absent: 0 = no card or card not seen
**Child’s age**	Age in months at time of survey
**Type of residence**	Coded as: 1 = urban; 2 = rural
**Women’s education**	Coded as: 1 = no education; 2 = incomplete primary; 3 = completed primary; 4 = incomplete secondary; 5 = completed secondary; 6 = higher education
**Wealth index**	Coded as: 1 = poorest; 2 = poorer; 3 = middle; 4 = richer; 5 = richest

ANC – antenatal care, HBR – home-based record, SBA – skilled birth attendance, PNC – postnatal care, MCH – maternal and child health, MCHHB – maternal and child health handbook, qANC – quality antenatal care, qPNC – quality postnatal care

### Statistical analysis

We merged the DHS data sets for pooled analysis. We conducted a logistic regression analysis to assess associations between HBR ownership and completion of the continuum of quality MCH care and estimated unadjusted and adjusted models. We also conducted subgroup analyses using separate logistic regression models for each MCH service component. Because these analyses were component-specific, we did not include interaction terms. The multivariable logistic regression controlled for known predictors of MCH service use: residence type, maternal education, and the household wealth index [23], and we included the county as a control variable. A multilevel model was unnecessary because ‘Has health card’ was only asked for the last-born child. For missing data, we conducted a complete-case analysis and used no imputation. We only included cases with complete data for qANC, qPNC, and qANC + SBA + qPNC. We reported the results as odds ratios (ORs) and adjusted ORs (aORs) with 95% confidence intervals (CIs).

We used IBM SPSS Statistics, version 29.0 (IBM Corp, Chicago, Illinois, USA) for all analyses. We used the complex samples package to consider the multistate sampling design, incorporating sample weights, primary sampling units, and strata [21]. We also applied country-level weights.

## RESULTS

### Participant characteristics

The analysis included 89 902 women and their youngest children. Among them, 37.7% of the women resided in urban areas, 21.4% were in the poorest quintile, and 35.1% had no formal education (**Table 2**).

**Table 2:** Characteristics of the study participants

	n (weighted %)
**Type of residence**	
Urban	33 909 (37.7)
Rural	55 993 (62.3)
**Country**	
Burkina Faso	1995 (2.2)
Côte d'Ivoire	2608 (2.9)
Kenya	4257 (1.7)
Senegal	1550 (4.7)
Indonesia	14 444 (16.1)
Timor-Leste	89 (0.1)
Ethiopia	9684 (10.8)
Gambia	215 (0.2)
Guinea	1585 (1.8)
Liberia	437 (0.5)
Mali	2504 (2.8)
Mauritania	433 (0.5)
Maldives	22 (0.0)
Nigeria	19 975 (22.2)
Pakistan	18 538 (20.6)
Tanzania	6332 (7.0)
South Africa	3870 (4.3)
Zimbabwe	1363 (1.5)
**Sex of the child**	
Male	45 750 (50.9)
Female	44 153 (49.1)
**Wealth index**	
Poorest	19 238 (21.4)
Poorer	18 665 (20.8)
Middle	18 212 (20.3)
Richer	17 381 (19.3)
Richest	16 407 (18.2)
**Mother’s educational attainment**	
No education	31 543 (35.1)
Incomplete primary	8934 (9.9)
Complete primary	11 856 (13.2)
Incomplete secondary	14 900 (16.6)
Complete secondary	13 855 (15.4)
Higher	8815 (9.8)

### HBR ownership by country

Among participants, 18.6% owned an MCHHB, 42.2% owned another HBR type, and 39.1% reported having no HBR. Ownership rates varied considerably in countries where the MCHHB is distributed nationally. Notably, the MCHHB ownership was relatively low in Indonesia (58.5%) and Timor-Leste (51.9%).

Among countries that have not yet implemented the MCHHB, the proportion of women owning any HBR type varied widely, from 90.1% in the Gambia to 40.8% in Nigeria (**Table 3**).

**Table 3:** Types of HBR ownership by country*

	MCHHB	HBR, not MCHHB	No HBR
**Total**	16 766 (18.6)	37 958 (42.2)	35 178 (39.1)
**Burkina Faso**	1730 (86.7)		265 (13.3)
**Côte d'Ivoire**	2049 (78.6)		559 (21.4)
**Kenya**	3213 (75.5)		1044 (24.5)
**Senegal**	1271 (82.0)		279 (18.0)
**Indonesia**	8457 (58.5)		5988 (41.5)
**Timor-Leste**	46 (51.9)		43 (48.1)
**Ethiopia**		6838 (70.6)	2846 (29.4)
**The Gambia**		194 (90.1)	21 (9.9)
**Guinea**		1041 (65.6)	545 (34.4)
**Liberia**		280 (64.0)	157 (36.0)
**Mali**		1458 (58.2)	1046 (41.8)
**Mauritania**		197 (45.4)	237 (54.6)
**Maldives**		18 (81.8)	4 (18.2)
**Nigeria**		8153 (40.8)	11 822 (59.2)
**Pakistan**		11 069 (59.7)	7469 (40.3)
**Tanzania**		4962 (78.4)	1370 (21.6)
**South Africa**		2691 (69.5)	1179 (30.5)
**Zimbabwe**		1058 (77.6)	305 (22.4)

MCHHB – maternal and child health handbook, HBR – home-based record

*Variables are presented as numbers (weighted percentages).

### Service utilisation by HBR ownership

**Figure 1:**
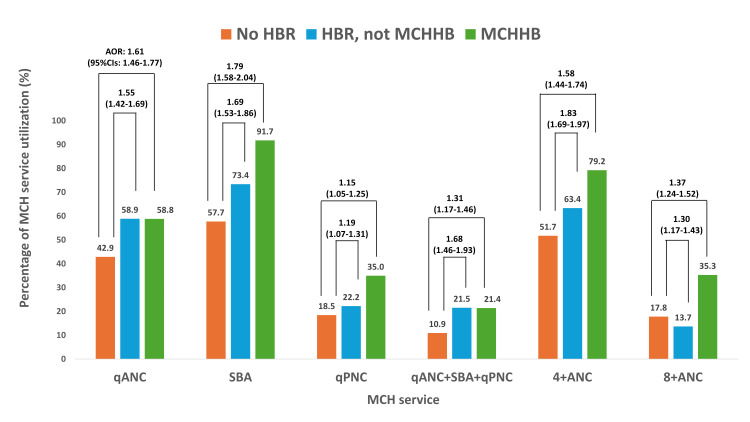
MCH service utilisation by types of HBRs and the association between HBR ownership and MCH service utilisation. AOR – adjusted odds ratio, CI – confidence interval, HBR – home-based record, MCH – maternal and child health, MCHHB – maternal and child health handbook, SBA – skilled birth attendance, qANC – quality antenatal care, qPNC – quality postnatal care, 4+ANC – at least four antenatal care visits, 8+ANC – at least eight antenatal care visits.

Among women who achieved qANC, 42.9% had no HBR, 58.9% owned an HBR other than an MCHHB, and 58.8% owned an MCHHB (**Figure 1**). Among those without HBR, 57.7% received SBA and 18.5% qPNC. On the other hand, among those with HBR other than MCHHB, 73.4% received SBA and 22.2% qPNC, and among those with MCHHB, 91.7% received SBA and 35.0% qPNC.

Furthermore, 51.7% of women without an HBR attended at least four ANC visits, compared with 64.3% of those with HBR and 79.2% of those with an MCHHB.

### HBR ownership and MCH service utilisation

Women with MCHHB (OR = 1.90; 95% CI = 1.75–2.06) or another type of HBR (OR = 1.91; 95% CI = 1.76–2.06) were significantly more likely to achieve qANC compared with women without an HBR (**Figure 1**; Table S1 in the **Online Supplementary Document**). Likewise, women with MCHHB (OR = 8.14; 95% CI = 7.27–9.12) or another HBR (OR = 2.02; 95% CI = 1.85–2.20) were more likely to deliver with an SBA. Additionally, women with MCHHB (OR = 2.38; 95% CI = 2.18–2.59) or another HBR (OR = 1.26; 95% CI = 1.16–1.37) were more likely to receive qPNC.

Women who owned an MCHHB (OR = 2.22; 95% CI = 2.01–2.44) or another HBR (OR = 2.23; 95% CI = 2.00–2.49) were more likely to consistently utilise qANC, SBA, and qPNC. In terms of ANC visit frequency, MCHHB owners had significantly higher odds of attending at least four visits (OR = 3.56; 95% CI = 3.28–3.87) and more than eight visits (OR = 2.52; 95% CI = 2.31–2.75). Women with HBR were more likely to attend four visits (OR = 1.61; 95% CI = 1.50–1.74) but less likely to attend more than eight (OR = 0.73; 95% CI = 0.66–0.81).

The adjusted multivariable logistic regression showed similar trends. Women with an MCHHB (aOR = 1.61; 95% CI = 1.46–1.77) and another HBR (aOR = 1.55; 95% CI = 1.42–1.69) were significantly more likely to achieve qANC. Also, those with MCHHB (aOR = 1.79; 95% CI = 1.58–2.03) and another HBR (aOR = 1.69; 95% CI = 1.53–1.86) were more likely to utilise SBA. Additionally, women with an MCHHB (aOR = 1.15; 95% CI = 1.05–1.25) or other HBRs (aOR = 1.19; 95% CI = 1.07–1.31) had modestly increased odds of service use.

### Association between MCHHB and other HBR types and MCH service utilisation

We conducted logistic regressions, including multivariable models, to assess differences in the associations between MCHHB and other HBR types and MCH service utilisation. We adjusted these models for type of residence, wealth index, women’s educational attainment, and country (Table S2 in the **Online Supplementary Document**).

Compared to those owning other HBR types, women who owned an MCHHB were significantly more likely to deliver with an SBA (OR = 4.03; 95% CI = 3.57–4.56), have higher qPNC (OR = 1.88; 95% CI = 1.73–2.05), attend at least four ANC visits (OR = 2.21; 95% CI = 2.02–2.41), and attend more than eight ANC visits (OR = 3.44; 95% CI = 3.08–3.85).

However, no significant differences were observed between the two groups in achieving qANC or continuous utilisation of qANC, SBA, and qPNC. Furthermore, after adjusting for residence type, wealth index, women’s educational attainment and country, the associations were no longer significant.

### Association between HBR ownership and MCH service components

Compared to those without an HBR, women with MCHHB were more likely to receive blood pressure measurement (OR = 4.39; 95% CI = 3.60–5.35), urine testing (OR = 1.76; 95% CI = 1.62–1.91), blood testing (OR = 1.63; 95% CI = 1.50–1.78), and iron supplementation (OR = 6.11; 95% CI = 5.49–6.82) during ANCs. Women with other HBR types had lower odds of blood pressure measurement (OR = 0.29; 95% CI = 0.25–0.34) but higher odds of urine testing (OR = 2.32; 95% CI = 2.03–2.65), blood testing (OR = 5.05; 95% CI = 4.36–5.84), and iron supplementation (OR = 1.83; 95% CI = 1.67–2.00).

For PNC, women who owned MCHHB were more likely to receive cord examination (OR = 2.95; 95% CI = 2.70–3.23), newborn temperature measurement (OR = 2.89; 95% CI = 2.65–3.15), counselling on newborn danger signs (OR = 2.40; 95% CI = 2.21–2.61), counselling on breastfeeding (OR = 2.59; 95% CI = 2.38–2.81), and observed breastfeeding (OR = 2.55; 95% CI = 2.35–2.77). Women with other HBRs also had higher odds of these PNC services, although the estimates were generally smaller.

**Figure 2:**
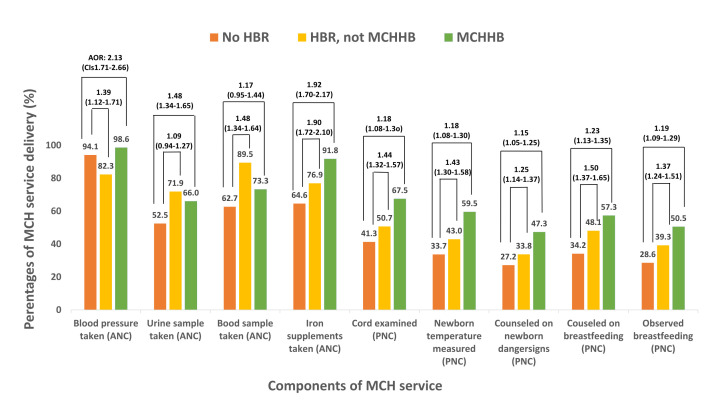
Association between HBR ownership and each component of MCH service utilisation. ANC – antenatal care, AOR – adjusted odds ratio, CI – confidence interval, HBR – home-based record, MCH – maternal and child health, MCHHB – maternal and child health handbook, PNC – postnatal care.

The adjusted analyses showed similar patterns. Compared to those without HBR, women with other HBRs were more likely to receive blood pressure measurement (aOR = 1.39; 95% CI = 1.12–1.71) and iron supplementation (aOR = 1.90; 95% CI = 1.72–2.10) (**Figure 2**; Table S3 in the **Online Supplementary Document**). However, the associations for urine testing (aOR = 1.09; 95% CI = 0.94–1.27) and blood testing (aOR = 1.17; 95% CI = 0.95–1.44) were not statistically significant.

For PNC, MCHHB ownership continued to be associated with cord examination (aOR = 1.18; 95% CI = 1.08–1.30), newborn temperature measurement (aOR = 1.18; 95% CI = 1.08–1.29), counselling on danger signs (aOR = 1.15; 95% CI = 1.05–1.25), counselling on breastfeeding (aOR = 1.23; 95% CI = 1.13–1.35), and observed breastfeeding (aOR = 1.19; 95% CI = 1.09–1.29). Ownership of other HBRs was also associated with higher adjusted odds of PNC services.

## DISCUSSION

We examined associations between owning HBRs and the continuum of quality MCH care, examining differences between owning MCHHB and other HBR types. The findings suggest that mothers with any HBR type, including MCHHB, may receive improved continuous and quality care than those without any HBR. In addition, the MCHHB may facilitate ANC service provision more effectively than other HBRs. Although HBRs are widely adopted, to our knowledge, we are the first to evaluate their association with the continuum of quality MCH care using pooled, nationally representative multi-country DHS data, suggesting that the findings may be broadly relevant across diverse LMIC settings.

We found that women who owned any HBR type, including MCHHB, were more likely to attend at least four and more than eight ANC visits than women without an HBR, aligning with previous studies demonstrating a positive association between HBR ownership and attending at least four ANC visits [18,24], as well as WHO guidelines recognising HBRs as means for promoting continuity of care [16]. However, few studies have examined the association between HBR ownership and attendance of at least eight ANC visits, despite the WHO’s updated recommendation [11]. Our findings contribute to this under-researched area.

Although we found no significant differences between the MCHHB and other HBR types, owning any HBR type was associated with increased utilisation of MCH services, including SBA and PNC. Most studies have investigated associations between HBRs and individual care components, and our results align with those reporting positive associations between HBR ownership and maternal tests and education [17], maternal care [25], and the positive impact of MCHHB on delivery with SBA [18]. Some studies have described associations between HBRs and newborn care, such as immediate breastfeeding [24,26], but few examined HBRs and PNC. Therefore, our finding of a positive association between HBR ownership and postnatal care contributes to the literature.

Women more engaged with health services may be more likely to retain and present HBRs, complicating causal interpretation. Although our cross-sectional design limits causal inference, evidence from countries where the MCHHB was rolled out prior to data collection supports the assumed temporal ordering. Future studies employing longitudinal designs, instrumental-variable methods, or natural experiments are needed to clarify causal relationships.

Although we did not identify significant differences between owning MCHHB and other HBR types regarding continuous care utilisation and quality, we found that mothers who owned MCHHB were more likely to have urine and blood tests than those without HBR, suggesting the importance of owning an HBR that covers ANC. The MCHHB is distinguished by its comprehensive documentation, covering care from pregnancy to five years postpartum and providing detailed information on safe pregnancy, delivery, and child development. It may thus serve as a reminder for healthcare providers. Although many countries have adopted child vaccination cards, HBRs are not always available for ANC. Furthermore, while general HBR ownership may encourage initial visits to health facilities [27], continued engagement depends on women’s understanding of the value of the records and their prior care experiences [23,28].

The MCHHB provides structured guidance to healthcare providers regarding expected care at each ANC contact. Notably, health workers’ knowledge, attitudes, and incentives, along with the facility environment, can also influence care quality. Provider factors, including knowledge, significantly affect ANC quality in MCHHB settings [29]. In some cases, MCHHB is distributed without explanation, reducing its role to mere possession [30]. Qualitative studies exploring provider behaviour, perceptions, and constraints in real-world cases are needed to clarify these dynamics. Further studies, particularly those using qualitative methods, are needed to evaluate how MCHHB is used in practice and how provider-related factors shape its impact.

Following multivariable regression, most univariate associations, consistent with the descriptive statistics, changed direction. These findings suggest that maternal educational attainment and household wealth substantially influence care uptake and MCHHB possession. The MCHHB may require a certain level of literacy and understanding for effective use, although it often includes rich visual content to accommodate varying literacy levels. Therefore, mothers with higher education may derive greater benefit from MCHHBs, whereas vaccination cards may be more accessible to mothers with lower education levels.

Although we included countries where the MCHHB has been officially adopted and distributed nationally, multiple HBR formats coexist. In Côte d’Ivoire, an alternative MCHHB version for Muslim families is available, but it contains fewer informational pages. Additionally, simpler HBR forms are still used. Such variations result in reduced learning opportunities and knowledge gaps, potentially influencing MCH service utilisation.

Moreover, HBRs differ in form, from ANC or vaccination cards to comprehensive ‘mother’s booklets’, with overlapping content. These similarities may overestimate the impact of non-MCHHB HBRs on service use and quality, potentially contributing to the lack of significance in the differences across record types. Future research should consider a more detailed HBR classification to accurately assess their effects.

Finally, our findings suggest that MCHHBs may enhance service delivery by reminding healthcare providers of appropriate care. However, care quality may vary depending on provider-related factors, such as knowledge and motivation. Thus, provider training should be an integral component of MCHHB programmes, alongside qualitative assessments to understand challenges in adopting and delivering quality care. Moreover, the coexistence of multiple HBR formats may hinder effective use and increase provider workload. Countries with such diversity may consider adopting a comprehensive HBR that includes ANC components, as our results indicate improved uptake of urine and blood testing when implemented with a training package.

### Limitations

This study has several limitations. First, provider characteristics may influence care quality, but we could not include provider-level factors because we lacked relevant variables. Second, we could not account for differences in HBR form and content, potentially leading to an overestimation of MCHHB and HBR ownership. Moreover, variations in content, format, and programmatic implementation across countries and survey years, as well as temporal changes, may limit the comparability of the ‘card seen’ measure. Such heterogeneity should be considered when interpreting pooled estimates. Third, we excluded some important components due to missing data in specific data sets, such as tetanus vaccination, which was excluded from the qANC indicator in all countries because it was unavailable in Indonesia and Timor-Leste. Additionally, recall bias may have influenced the data, and differences in interview timing may have affected card availability, as mothers reported events that occurred up to three years before data collection. We assessed this potential bias by restricting the analysis to mothers and children whose deliveries or births occurred within two months of data collection; the direction and magnitude of the results did not differ from the main analysis (data not shown). Furthermore, we focused on MCHHB/HBR possession for the most recent birth. When restricted to mothers who owned an MCHHB or other HBR for their firstborn child, the results were consistent with those of the main analysis (data not shown). Finally, because the DHS is cross-sectional, we could not establish causal relationships, and reverse causality remains a possibility.

## CONCLUSIONS

We found that mothers who possessed any type of HBR were more likely to consistently utilise quality MCH services. Meanwhile, there were no significant differences between HBR type and the continuity of quality MCH care for MCHHBs, but MCHHB ownership was significantly associated with improved uptake of urine and blood tests.

Although MCHHB may enhance maternal understanding of ANC and assist providers in delivering appropriate care, our analysis was limited to HBR ownership and did not capture how the records were used. Furthermore, we did not consider provider-level factors or variations in content and format, highlighting the need for further research to examine the impact of HBR functionality and use across diverse settings.

## Additional material


Online Supplementary Document
