# Supplementary material for: Association between the ownership of home-based records and continuous, quality maternal and child health service utilisation: a multi-country analysis of Demographic Health Surveys from 18 low- and middle-income countries
Source: J Glob Health. 2026 Feb 13;16:04052. doi: 10.7189/jogh.16.04052 (PMC12900547; doi:10.7189/jogh.16.04052)
Supplement: Online Supplementary Document [file jogh-16-04052-s001.pdf]

Supplement to: Saito A, Kondo M. Association between the ownership of home-based records and continuous, quality maternal and child health service utilisation: a multi-country analysis of Demographic Health Surveys from 18 low- and middle-income countries. J Glob Health. 2026;16:04052.

Table S1. Association between HBR ownership and MCH service utilization

	Number of women/child receive	%	Number of women/child not receive	%	OR	95%CI		AOR	95%CI	
**HBR**				**qANC**						
No HBR	14696	42.9	19573	57.1	Ref.			Ref.		
HBR, not MCHHB	21774	58.9	15217	41.1	1.91	1.76	2.06	1.55	1.42	1.69
MCHHB	9790	58.8	6855	41.2	1.90	1.75	2.06	1.61	1.46	1.77
				**SBA**						
No HBR	20283	57.7	14848	42.3	Ref.			Ref.		
HBR, not MCHHB	27825	73.4	10093	26.6	2.02	1.85	2.20	1.69	1.53	1.86
MCHHB	15320	91.7	1378	8.3	8.14	7.27	9.12	1.79	1.58	2.03
				**qPNC**						
No HBR	6391	18.5	28236	81.5	Ref.			Ref.		
HBR, not MCHHB	8423	22.2	29492	77.8	1.26	1.16	1.37	1.19	1.07	1.31
MCHHB	5294	35.0	9847	65.0	2.38	2.18	2.59	1.15	1.05	1.25
				**qANC+SBA+qPNC**						
No HBR	3792	10.9	30883	89.1	Ref.			Ref.		
HBR, not MCHHB	8120	21.5	29614	78.5	2.23	2.00	2.49	1.68	1.46	1.93
MCHHB	3301	21.4	12118	78.6	2.22	2.01	2.44	1.31	1.17	1.46
				**4+ANC**						
No HBR	18170	51.7	16959	48.3	Ref.			Ref.		
HBR, not MCHHB	24026	63.4	13892	36.6	1.61	1.50	1.74	1.83	1.69	1.97
MCHHB	13230	79.2	3467	20.8	3.56	3.28	3.87	1.58	1.44	1.74
				**8+ANC**						
No HBR	6260	17.8	28869	82.2	Ref.			Ref.		
HBR, not MCHHB	5187	13.7	32731	86.3	0.73	0.66	0.81	1.30	1.17	1.43
MCHHB	5896	35.3	10801	64.7	2.52	2.31	2.75	1.37	1.24	1.52

OR: Odds ratio; AOR: Adjusted odds ratio; 95%CI: 95% confidential interval;HBR: home-based record; MCHHB: maternal and child health handbook; qANC: quality antenatal care; SBA: skilled birth attendant; qPNC: quality postnatal care; 4+ANC: at least 4 antenatal care visits; 8+ANC: at least 8 antenatal care visits; Ref.: reference.

Table S2. Differences in the association of various types of HBRs with MCH service utilization

	Number of women/child receive	%	Number of women/child not receive	%	OR	95%CI		AOR	95%CIs	
**HBR**					**qANC**					
HBR, not MCHHB	21774	58.9	15217	41.1		Ref.			Ref.	
MCHHB	9790	58.8	6855	41.2	0.10	0.91	1.10	1	1	1
					**SBA**					
HBR, not MCHHB	27825	73.4	10093	26.6		Ref.			Ref.	
MCHHB	15320	91.7	1378	8.3	4.03	3.57	4.56	1	1	1
					**qPNC**					
HBR, not MCHHB	8423	22.2	29492	77.8		Ref.			Ref.	
MCHHB	5294	35.0	9847	65.0	1.88	1.73	2.05	1	1	1
					**qANC+SBA+qPNC**					
HBR, not MCHHB	8120	21.5	29614	78.5		Ref.			Ref.	
MCHHB	3301	21.4	12118	78.6	0.99	0.90	1.10	1	1	1
					**4+ANC**					
HBR, not MCHHB	24026	63.4	13892	36.6		Ref.			Ref.	
MCHHB	13230	79.2	3467	20.8	2.21	2.02	2.41	1	1	1
					**8+ANC**					
HBR, not MCHHB	5187	13.7	32731	86.3		Ref.			Ref.	
MCHHB	5896	35.3	10801	64.7	3.44	3.08	3.85	1	1	1

OR: Odds ratio; AOR: Adjusted odds ratio; 95%CI: 95% confidential interval;HBR: home-based record; MCHHB: maternal and child health handbook; qANC: quality antenatal care; SBA: skilled birth attendant; qPNC: quality postnatal care; 4+ANC: at least 4 antenatal care visits; 8+ANC: at least 8 antenatal care visits; Ref.: reference.

Table S3. Association between HBR ownership and each component of MCH service utilization

	Number of women/child receive	%	Number of women/child not receive	%	OR	95%CI		AOR	95%CI	
**Blood pressure taken (ANC)**										
No HBR	8817	94.1	554	5.9		Ref			Ref	
HBR, not MCHHB	4723	82.3	1016	17.7	0.29	0.25	0.34	1.39	1.12	1.71
MCHHB	14976	98.6	214	1.4	4.39	3.60	5.35	2.13	1.71	2.66
**Urine sample taken (ANC)**										
No HBR	4914	52.5	4451	47.5		Ref			Ref	
HBR, not MCHHB	4126	71.9	1613	28.1	2.32	2.03	2.65	1.09	0.94	1.27
MCHHB	10026	66.0	5161	34.0	1.76	1.62	1.91	1.48	1.34	1.65
**Blood sample taken (ANC)**										
No HBR	5872	62.7	3495	37.3		Ref			Ref	
HBR, not MCHHB	5134	89.5	605	10.5	5.05	4.36	5.84	1.17	0.95	1.44
MCHHB	11125	73.3	4058	26.7	1.63	1.50	1.78	1.48	1.34	1.64
**Iron supplements taken (ANC)**										
No HBR	22689		12441	35.4		Ref			Ref	
HBR, not MCHHB	29166		8752	23.1	1.83	1.67	2.00	1.90	1.72	2.10
MCHHB	15325		1374	8.2	6.11	5.49	6.82	1.92	1.70	2.17
**Cord examined (PNC)**										
No HBR	14304	35.4	20326	58.7		Ref			Ref	
HBR, not MCHHB	19233	23.1	18682	49.3	1.46	1.35	1.58	1.44	1.32	1.57
MCHHB	10219	8.2	4922	32.5	2.95	2.70	3.23	1.18	1.08	1.30
**Newborn temperature measured (PNC)**										
No HBR	11683	33.7	22947	66.3		Ref			Ref	
HBR, not MCHHB	16287	43.0	21629	57.0	1.48	1.37	1.60	1.43	1.30	1.58
MCHHB	9017	59.5	6125	40.5	2.89	2.65	3.15	1.18	1.08	1.29
**Counseled on newborn danger signs (PNC)**										
No HBR	9412	27.2	25211	72.8		Ref			Ref	
HBR, not MCHHB	12802	33.8	25114	66.2	1.37	1.27	1.47	1.25	1.14	1.37
MCHHB	7162	47.3	7980	52.7	2.40	2.21	2.61	1.15	1.05	1.25
**Counseled on breastfeeding (PNC)**										
No HBR	11832	34.2	22798	65.8		Ref			Ref	
HBR, not MCHHB	18242	48.1	19674	51.9	1.79	1.65	1.93	1.50	1.37	1.65
MCHHB	8680	57.3	6461	42.7	2.59	2.38	2.81	1.23	1.13	1.35
**Observed breastfeeding (PNC)**										
No HBR	9905	28.6	24720	71.4		Ref			Ref	
HBR, not MCHHB	14887	39.3	23029	60.7	1.61	1.49	1.75	1.37	1.24	1.51
MCHHB	7654	50.5	7488	49.5	2.55	2.35	2.77	1.19	1.09	1.29

OR: Odds ratio; AOR: Adjusted odds ratio; 95%CI: 95% confidential interval; HBR: home-based record; MCHHB: maternal and child health handbook; Ref.: reference.

international society of global health

Author checklist – Journal of Global Health (JoGH)

Please check the corresponding item when completed.

	YES	NO	N/A
**Does your title page contain all necessary content?**			
Contains article title.	✔	☐	☐
Contains author names, surnames, ORCIDs, and affiliations.	✔	☐	☐
Contains indications relating to joint first/senior authorship or equal contribution.	☐	☐	✔
Contains a structured abstract of up to 300 words with Background, Methods, Results, Conclusions, and Registration (for original research articles).	✔	☐	☐
Contains an unstructured abstract of up to 200 (for viewpoints) or 400 words (for editorials)	☐	☐	✔
Contains keywords (up to six, separated with semicolon).	✔	☐	☐
Contains two key messages (only for viewpoints).	☐	☐	✔
**Is your main text complete?**			
Has up to 3500 words (for original research articles)/5000 words (for qualitative research or reviews)/1500 words (for viewpoints)/3000 words (for editorials)	✔	☐	☐
Contains Background, Methods, Results, Discussion, and Conclusions sections (for original research articles).	✔	☐	☐
Contains all relevant tables and figures, with the figures sent separately as TIF files at least 600 DPI in quality.	✔	☐	☐
Contains properly formatted references with formatted, sequentially numbered in-text citations in square brackets.	✔	☐	☐
Contains supplement as PDF/Excel files, with datasets deposited in repository.	✔	☐	☐
